# Liposarcoma of the Spermatic Cord: Impact of Final Surgical Intervention—An Institutional Experience

**DOI:** 10.1155/2016/4785394

**Published:** 2016-04-14

**Authors:** R. Bachmann, J. Rolinger, P. Girotti, H. G. Kopp, K. Heissner, B. Amend, A. Königsrainer, R. Ladurner

**Affiliations:** ^1^Department of General, Visceral and Transplant Surgery, University Hospital Tübingen, Hoppe-Seylerstrasse 3, 72076 Tübingen, Germany; ^2^Department of Oncology II, University Hospital Tübingen, Otfried-Müller-Strasse 10, 72076 Tübingen, Germany; ^3^Department of Urology, University Hospital Tübingen, Hoppe-Seylerstrasse 3, 72076 Tübingen, Germany

## Abstract

*Background*. Paratesticular liposarcomas are almost always mistakenly diagnosed as inguinal hernias subsequently followed by inadequate operation.* Methods*. 14 consecutive patients with paratesticular liposarcoma were retrospectively reviewed. Preoperative management was evaluated. Disease-free and overall survival were determined.* Results*. In 11 patients primary and in 3 patients recurrent liposarcoma of the spermatic cord were diagnosed. Regarding primary treatment in primary surgical intervention resection was radical (R0) in 7 of 14 (50%) patients, marginal (R1) in 6 (43%) patients, and incomplete with macroscopic residual tumour (R2) in 1 (7%) patient. Primary treatment secondary surgical intervention was performed in 4 patients: resection was radical (R0) in 3 (75%) patients and marginal (R1) in 1 (25%) patient. Regarding secondary treatment in recurrent disease resection was marginal (R1) in 3 patients (100%). Final histologic margins were negative in 10 patients with primary disease (71%) and positive in 4 patients with subsequent recurrent disease. After radical resection disease-free survival rates at 3 years were 100%. Overall survival at 4.5 years (54 (18–180) months) was 64%.* Conclusion*. An incomplete first surgical step increases the number of positive margins leading to local recurrences and adverse prognoses. Aggressive surgery should be attempted to attain 3-dimensional negative margins.

## 1. Introduction

Sarcomas of the paratesticular region are rare. The most common histologic subtypes for adult paratesticular malignancies are liposarcoma, leiomyosarcoma, and rhabdomyosarcoma. Initially misdiagnosed as inguinal, scrotal hernia or lipoma of the spermatic cord the primary treatment is therefore only marginal excision. Primary or subsequent radical inguinal orchiectomy with spermatic cord resection is the standard surgical approach, permitting the longest local and systemic disease-free survival. A wide circumferential resection margin, especially needed in the case of liposarcoma, can rarely be achieved in these cases. Compartmental surgery as an alternative is mutilating and difficult due to anatomical constraints. However, it should be emphasized that the best results in terms of the local control of recurrences, according to the literature, are achieved with wide aggressive surgery with a simultaneous ipsilateral pelvic and inguinal lymphadenectomy. Additional adjuvant radiation has been proposed from small retrospective studies [1–3] to reduce the rate of local recurrence and to minimize surgical resection. Significant risk factors for tumour recurrence and progression have been identified in adult sarcoma and include histological subtypes, tumour size, grade, local invasion, lymph node involvement, and surgical margin status. Exclusive to the liposarcoma subgroup we see quality of surgical margins affecting local outcome, while negative surgical margins become directly associated with disease-specific survival [4].

The purpose of our retrospective study was to investigate the treatment of patients in the initial presenting of disease, the incidence of recurrence, treatment in recurrence, and the overall survival in our own patients with liposarcoma of the spermatic cord.

## 2. Patients and Methods

The study is a retrospective analysis of 14 consecutive patients who presented at our hospital between December 2006 and July 2013 with primary or local recurrent diagnosis of liposarcoma of the spermatic cord. All clinical information of sarcoma was retrieved from the archives of the University Hospital Tübingen and telephone inquiries to primary treating physicians. Primary admission was the department of general, visceral and transplantation surgery in 5 patients, department of urology in 7 patients, and the cardiothoracic and a tertiary teaching hospital with 1 patient each.

Primary disease was defined as presentation to our hospital at time of initial diagnosis including patients with initial marginal resection. Recurrent disease was defined as presentation to our hospital with locally recurrent disease after initial final resection.

Preoperative management was evaluated using the electronic database. Adequate surgical resection was evaluated based on the pathology report, the surgical report of the referring surgeon, and a radiographic investigation.

Disease-free survival and overall survival were determined. Recurrence-free survival (RFS) was defined from date of final resection to date of any recurrence. Overall survival (OS) was defined from date of final resection to date of death.

## 3. Results

### 3.1. Clinical Characteristics

During the study period, 14 patients with localized paratesticular liposarcoma were matching the inclusion criteria. Median age at presentation was 67 years (range, 48–86 years). A total of 11 (79%) patients presented to the University Hospital Tübingen with primary disease; of these 6 patients had their initial operation in the University Hospital Tübingen and 5 patients had initial R1 resection at an outside hospital incidentally after repair of an inguinal hernia or inguinal orchiectomy. Three patients (21%) were admitted with recurrent disease. See Table 1.

Four of the patients presented with a second malignant disease, two of them with prostate cancer, one with B-cell lymphoma, and the last with a locally advanced rectal cancer.

### 3.2. Preoperative Evaluation

Physical examination, revealing a swelling around the spermatic cord, soft in consistency and movable with the spermatic cord and/or testis, was reported in all patients. Ultrasound was generally performed at first in all patients. It typically found a mass around the spermatic cord and/or testis. Size of the soft tissue mass median was 7.9 (3–11) cm with the site in 9 cases on the left and in 5 cases on the right. Three patients had a CT-scan before their first surgery and 11 patients after the established diagnosis. Immediately after first resection of the inguinoscrotal mass the local radiological finding was negative in these 11 cases, and no lymph node or distant metastasis was described. An MR-scan was used in one patient before the first surgical approach and in another after the surgery.

### 3.3. Surgical Treatment

In primary disease first surgical intervention tumour resection was performed with resection of tumour [5] with spermatic cord resection [2] and orchiectomy [6]. The final surgical treatment in primary disease desired after initial marginal resection wide circumferential reresection in 5 patients with spermatic cord resection (3 patients), lymph node dissection (2 patients), resection of soft tissue mass (5 patients), and orchiectomy (1 patient) and resection and reconstruction of iliac artery [4]. See Table 3.

In recurrent disease the surgical treatment desired abdominal wall resection, Gore-Tex^©^ reconstruction instead of peritoneum and prolene-prosthesis as muscle replacement and regional lymphadenectomy [4], and multivisceral resection [1]. See Tables 2, 3, and 4.

Regarding histological workup the sarcomas were highly differentiated in 7 (G1) and well differentiated (G2) in the other 7 patients.

### 3.4. Surgical Treatment Outcome

Regarding primary treatment in primary surgical intervention resection was radical (R0) in 7 of 14 (50%) patients, marginal (R1) in 6 (43%) patients, and incomplete with macroscopic residual tumour (R2) in 1 (7%) patient. Primary treatment secondary surgical intervention was performed in 4 patients: resection was radical (R0) in 3 (75%) patients and marginal (R1) in 1 (25%) patient. Regarding secondary treatment in recurrent disease resection was marginal (R1) in 3 patients (100%) and with macroscopic residual tumour (R2) in none of the patients. Final histologic margins were negative in 10 patients with primary disease (71%) and positive in 4 (29%) patients with subsequent recurrent disease. In secondary disease final histologic resection margins were marginal positive (R1) in all 3 operated patients.

### 3.5. Radiotherapy

Radiotherapy was performed in 3 patients after primary treatment (R0 = 2, R1 = 1) and 2 patients after secondary treatment (R1 = 2).

### 3.6. Survival Outcome

Nine of the 14 patients are alive without tumour on average for 51 (21–104) months after the first resection and histological diagnosis of liposarcoma of the spermatic cord. Five patients died, on average after 54 (18–180) months. The death of only one patient was related to the sarcoma. He was diagnosed as having a highly differentiated liposarcoma of the spermatic cord in 1998; after marginal resection of the primary tumour the first relapse occurred in 2006 and after five reresections he died in 2013 due to tumour-related complications.

## 4. Discussion

Herein we report clinical course and outcome for 14 patients with localized liposarcoma of the paratesticular compartment. Sarcomas of the genitourinary tract account for 2% of all urological tumours, 31% of all scrotal tumours, and for approximately 5% of all sarcomas in general. In terms of all paratesticular tumours, more than 75% of these lesions arise from the spermatic cord in adults [7]. Among the malignant tumours, the most common histotype is liposarcoma (46.4%), followed by leiomyosarcoma (20%), malignant fibrous histiocytomas (13%), and embryonal rhabdomyosarcoma (9%) [8]. Since primary diagnosis of paratesticular liposarcoma is difficult and patients usually present with a soft tissue mass in the right or left groin, the majority of patients with paratesticular liposarcoma are surgically supplied with presumed hernia by nonsarcoma specialists. In our series, about 60% of patients got primary treatment first surgical intervention at an extern hospital with marginal (43%) and intratumoural (7%) resection. At the same time in our patients the anamneses and clinical examination could have been able to indicate an unusual finding. Anamnestically described as slow growing, most asymptomatic solid mass and in clinical examination palpable mass, clearly distinguishable from the testis, not translucent like a hydrocele and not reducible like an inguinal hernia may have been warning. In this context, unusual intraoperative findings are better addressed by incisional biopsy to determine diagnosis as oncologic suboptimal resection.

A positive surgical margin seems to be the main risk factor for early local recurrence and distant metastasis, and a simple excision is suboptimal, as repeat wide excision has demonstrated microscopic residual disease in 27% of apparently complete excision [5]. The established treatment of soft tissue sarcoma should be a wide en bloc excision of all potentially contaminated tissues and to combine surgery with irradiation, even in those patients with a completely excised tumour. Consensus is found in the limited publications on the topic endorsing the role of radiotherapy in the adjuvant setting in paratesticular liposarcoma, especially for those patients who have already had one recurrence [9]. In our patient group, only two patients received radiotherapy: one after marginal resection of a third recurrence in our hospital and another before multivisceral resection in another hospital due to tumour recurrence.

The size of sarcomas can be considered an anatomical feature that influences the difficulty in achieving radical resection, especially in cases of a tumour occurring in sites with anatomical constraint limitations. Many authors report an extremely high rate of positive surgical margins or nonsatisfactory surgical results that lead to high rates of local failure; the risk of positive surgical margins is higher especially in the case of recurrence or large primary presentation. Nonetheless, the anatomic site is indeed an important prognostic factor in sarcoma, and the prognosis for spermatic cord tumours as well as for retroperitoneal sarcoma is considerably worse than for extremity tumours [10]. More than 59.2% of the patients in a study on retroperitoneal sarcoma by Zhao et al. [11] had tumours with diameters ≥20 cm. Several authors have suggested that larger tumours have a worse prognosis [12, 13]. This has been suggested primarily because large tumours frequently compress and/or infiltrate surrounding structures and tissues, which can significantly complicate complete resections, resulting in leftover microscopic residual disease. In our patients size of the soft tissue mass median was 7.9 (3–11) cm. However, several other studies [14, 15] have contradicted these findings by showing that there is no significant correlation between tumour size and survival prognosis. Also in our retrospective analysis, we did not observe an effect of tumour size on the overall survival of the patients. Rather than the tumour size, a complete resection seemed to influence the postoperative tumour-free survival rate in our patients. In our series the patients with negative margins, even if resected twice after marginal resection in an outside hospital, are alive without recurrence at a median of 51 (21–104) months after primary resection. The only initial radical resected patient in the deceased group died because of a B-cell lymphoma, while the second patient in this group with R0 resection in a second intervention with vascular replacement died because of pulmonary embolism. Tumour-related death occurred only in one patient. In the groin and also scrotum compartmental surgery is mutilating and therefore limited due to the factor of anatomy. Radical orchiectomy, resection of the soft tissue mass, and resection of the spermatic cord (inguinal approach) is the cornerstone of treatment in the management of this neoplasm, but the reported survival rates indicate the need for additional treatment [1]. Nevertheless, the use of adjuvant radiation also remains controversial. A high incidence of nodal metastasis is not seen in the histological workup of spermatic cord liposarcoma, meaning that adjuvant nodal treatment by radiation or wide lymph node dissection, associated with a higher rate of morbidity and postoperative complications, is not recommended. Finally, the benefits of adjuvant therapy after radical surgery is inconclusive in the literature due to the small numbers and the different histological subtypes summarized as spermatic cord sarcoma.

In the literature on retroperitoneal liposarcoma the overall 5- and 10-year survival rates were 36% and 14%, respectively. The locoregional relapse-free rate was 28% at five years and 9% at 10 years, and the distant relapse rate was 76% at five years and 60% at 10 years. For the patients undergoing radical resection, survival was 55% and 22% at five and 10 years, and the locoregional relapse rate was 50% and 18% at five and 10 years [6]. In the literature the actuarial local recurrence rate after primary radical resection is reported between 30% and 50% at 10 postoperative years [16]. In our patients (7 patients G1, 7 patients G2) we found no influence of tumour grading on survival and recurrence. The subsequent proposed treatment concept involves aggressive local surgical resection aiming at complete removal. Appropriate surgery consists of radical inguinal orchiectomy and wide excision of the potential prior surgical access and tumour bed including removal of all the soft tissues in the inguinal canal with ligation of the spermatic cord at the level of the interior inguinal canal. In disease extension through the proximal inguinal canal into the pelvis, removal of such disease along with resection of the lower abdominal wall with reconstruction is necessary. Regarding lymph nodal treatment we do not see the recommendation in paratesticular liposarcoma as nodal involvement is seldom. Regarding radiation therapy there is no indication in highly differentiated and well-differentiated paratesticular liposarcoma with complete resection. In case of positive resection margin radiation therapy is deferred because of the slow progression of disease. Concerning dedifferentiated and pleomorphic liposarcoma other rules are more accurate and radiation therapy is indicated prior to definitive resection [17].

## 5. Conclusion

Although this tumour is a rare lesion, it should be considered in the presentation of any inguinal or scrotal mass. Incorrect surgical intervention complicates definitive therapy and increases the risk of local-regional contamination and recurrence. Up to now radical inguinal orchiectomy, combined with wide resection of the soft tissue mass and the spermatic cord, seems to be the standard surgical approach in permitting the longest local and systemic disease-free survival.

## Figures and Tables

**Table 1:** Patient characteristics (*n* = 14).

Age (years)	67 (48–86)
Second malignancy, total	4
Prostate cancer	2
Rectal cancer	1
B-cell lymphoma	1
Size, median (cm)	7.9 (3–11)
Tumour site	9 left
	5 right
Ultrasound	14
CT-scan before first surgery	3
MRI-scan before first surgery	1
Preoperative biopsy	0
Sarcoma board before first surgery	0
Sarcoma board after first surgery	8

**Table 2:** Treatment details of primary resection (*n* = 14).

Resection of sarcoma and orchiectomy	7 (R1 = 2, R0 = 5)
Hernia repair, resection of sarcoma and inguinal spermatic cord	1 (R1)
Resection of sarcoma and spermatic cord	2 (R1 = 1, R0 = 1)
Resection of sarcoma	2 (R1 = 1, R0 = 1)
Hernia repair, resection of tumour	1 (R2)
Resection of sarcoma, spermatic cord, and sigma/rectum	1 (R1)

**Table 3:** Completion of primary R1 resection (*n* = 5).

Reresection, spermatic cord resection, and lymph node dissection	2 (R1 = 1, R0 = 1)
Reresection and orchiectomy	1 (R0)
Reresection	1 (R0)
Reresection, spermatic cord resection, and arterial reconstruction	1 (R0)

**Table 4:** Resection in the recurrence situation (*n* = 3).

Reresection, multivisceral resection (tumor, sigma ureter)	2 (R1)
Re-reresection (tumor, abdominal wall, and reconstruction)	1 (R1)

## References

[1] Blitzer P. H., Dosoretz D. E., Proppe K. H., Shipley W. U. (1981). Treatment of malignant tumors of the spermatic cord: a study of 10 cases and a review of the literature. *The Journal of Urology*.

[2] Fagundes M. A., Zietman A. L., Althausen A. F., Coen J. J., Shipley W. U. (1996). The management of spermatic cord sarcoma. *Cancer*.

[3] Catton C. N., Cummings B. J., Fornasier V., O'Sullivan B., Quirt I., Warr D. (1991). Adult paratesticular sarcomas: a review of 21 cases. *The Journal of Urology*.

[4] Radaelli S., Desai A., Hodson J. (2014). Prognostic factors and outcome of spermatic cord sarcoma. *Annals of Surgical Oncology*.

[5] Catton C., Jewett M., O'Sullivan B., Kandel R. (1999). Paratesticular sarcoma: failure patterns after definitive local therapy. *Journal of Urology*.

[6] Catton C. N., O'Sullivan B., Kotwall C., Cummings B., Hao Y., Fornasier V. (1994). Outcome and prognosis in retroperitoneal soft tissue sarcoma. *International Journal of Radiation Oncology, Biology, Physics*.

[7] Galosi A. B., Scarpelli M., Mazzucchelli R. (2014). Adult primary paratesticular mesenchymal tumors with emphasis on a case presentation and discussion of spermatic cord leiomyosarcoma. *Diagnostic Pathology*.

[8] Rodríguez D., Barrisford G. W., Sanchez A., Preston M. A., Kreydin E. I., Olumi A. F. (2014). Primary spermatic cord tumors: disease characteristics, prognostic factors, and treatment outcomes. *Urologic Oncology: Seminars and Original Investigations*.

[9] Littles J. F., Matter R. C., Herman G. (1992). Paratesticular liposarcoma: a report of two cases and review of the literature. *Journal of the National Medical Association*.

[10] Lewis J. J., Brennan M. F. (1996). Soft tissue sarcomas. *Current Problems in Surgery*.

[11] Zhao X. D., Li P., Huang X. H., Chen L., Liu N., She Y. G. (2015). Prognostic factors predicting the postoperative survival period following treatment for primary retroperitoneal liposarcoma. *Chinese Medical Journal*.

[12] An J. Y., Heo J. S., Noh J. H. (2007). Primary malignant retroperitoneal tumors: analysis of a single institutional experience. *European Journal of Surgical Oncology*.

[13] Stojadinovic A., Leung D. H. Y., Allen P., Lewis J. J., Jaques D. P., Brennan M. F. (2002). Primary adult soft tissue sarcoma: time-dependent influence of prognostic variables. *Journal of Clinical Oncology*.

[14] Brown R. E., St Hill C. R., Greene Q. J. (2011). Impact of histology on survival in retroperitoneal sarcomas. *American Journal of Surgery*.

[15] Mäkelä J., Kiviniemi H., Laitinen S. (2000). Prognostic factors predicting survival in the treatment of retroperitoneal sarcoma. *European Journal of Surgical Oncology*.

[16] Ballo M. T., Zagars G. K., Pisters P. W. T., Feig B. W., Patel S. R., Von Eschenbach A. C. (2001). Spermatic cord sarcoma: outcome, patterns of failure and management. *Journal of Urology*.

[17] Khandekar M. J., Raut C. P., Hornick J. L., Wang Q., Alexander B. M., Baldini E. H. (2013). Paratesticular liposarcoma: unusual patterns of recurrence and importance of margins. *Annals of Surgical Oncology*.

